# Global DNA Methylation Level in Tumour and Margin Samples in Relation to Human Papilloma Virus and Epstein–Barr Virus in Patients with Oropharyngeal and Oral Squamous Cell Carcinomas

**DOI:** 10.3390/biomedicines12040914

**Published:** 2024-04-20

**Authors:** Jadwiga Gaździcka, Krzysztof Biernacki, Karolina Gołąbek, Katarzyna Miśkiewicz-Orczyk, Natalia Zięba, Maciej Misiołek, Joanna Katarzyna Strzelczyk

**Affiliations:** 1 Department of Medical and Molecular Biology, Faculty of Medical Sciences in Zabrze, Medical University of Silesia in Katowice, 19 Jordana Street, 41-808 Zabrze, Polandjstrzelczyk@sum.edu.pl (J.K.S.); 2 Department of Otorhinolaryngology and Oncological Laryngology, Faculty of Medical Sciences in Zabrze, Medical University of Silesia in Katowice, 10 C. Skłodowskiej Street, 41-800 Zabrze, Poland

**Keywords:** global DNA methylation, head and neck squamous cell carcinoma (HNSCC), oral squamous cell carcinoma (OSCC), oropharyngeal squamous cell carcinoma (OPSCC), hypermethylation, tumour, margin, Epstein–Barr virus (EBV), human papilloma virus (HPV)

## Abstract

Background: Aberrant DNA methylation is a common epigenetic modification in cancers, including oropharyngeal squamous cell carcinoma (OPSCC) and oral squamous cell carcinoma (OSCC). Therefore, the analysis of methylation levels appears necessary to improve cancer therapy and prognosis. Methods: The enzyme-linked immunosorbent assay (ELISA) was used to analyse global DNA methylation levels in OPSCC and OSCC tumours and the margin samples after DNA isolation. HPV detection was conducted by hybridisation using GenoFlow HPV Array Test Kits (DiagCor Bioscience Inc., Hong Kong, China). EBV detection was performed using real-time PCR with an EBV PCR Kit (EBV/ISEX/100, GeneProof, Brno, Czech Republic). Results: OPSCC tumour samples obtained from women showed lower global DNA methylation levels than those from men (1.3% vs. 3.5%, *p* = 0.049). The margin samples from OPSCC patients with HPV and EBV coinfection showed global DNA methylation lower than those without coinfection (*p* = 0.042). G3 tumours from OSCC patients had significantly lower levels of global DNA methylation than G2 tumours (0.98% ± 0.74% vs. 3.77% ± 4.97%, *p* = 0.010). Additionally, tumours from HPV-positive OSCC patients had significantly lower global DNA methylation levels than those from HPV-negative patients (*p* = 0.013). In the margin samples, we observed a significant negative correlation between global DNA methylation and the N stage of OSCC patients (rS = −0.33, *p* = 0.039). HPV-positive OPSCC patients had higher global DNA methylation levels than HPV-positive OSCC patients (*p* = 0.015). Conclusion: We confirmed that methylation could be changed in relation to viral factors, such as HPV and EBV, as well as clinical and demographical parameters.

## 1. Introduction

DNA methylation is a common epigenetic modification catalysed by DNA methyltransferases (DNMTs) that transfer a methyl group to the cytosine residue and form 5-mC. The family of DNMTs includes DNMT1, which is important for copying the DNA methylation profile after replication. On the other hand, DNMT3A and DNMT3B are involved in de novo DNA methylation [1]. DNA methylation occurs in cytosine in the sequence of 5′-CpG-3′. However, CpG islands (CpGIs) are sequences with high densities of CpG, frequently located in the promoter region and unmethylated in normal somatic cells [2]. An aberrant DNA methylation profile may lead to different diseases, including cancer. Biomarkers of DNA methylation could be a potential diagnostic tool; however, further studies are warranted to standardise the methodologies [3].

Oropharyngeal squamous cell carcinoma (OPSCC) and oral squamous cell carcinoma (OSCC) are head and neck squamous cell carcinomas (HNSCCs). The main risk factors for HNSCC are tobacco and alcohol abuse, poor oral hygiene and infectious agents such as human papillomavirus (HPV) and Epstein–Barr virus (EBV) [4,5]. The mean survival rate has levelled off at 50% [5,6]. An increasing number of HPV-positive OPSCCs is being observed worldwide, where HPV-16 is the most dominant genotype [7] among the high-risk mucosal HPV types [8]. Moreover, HPV-positive HNSCC patients have far more favourable prognoses than HPV-negative HNSCC individuals [4,5]. The HPV status in HNSCC patients also has some influence on aberrant methylation [9]. EBV is an oncogenic human virus infecting more than 90% of the human adult population [10] and is associated with the risk of OSCC [11]. EBV can use multiple epigenetic modifications, including DNA methylation, to reprogram the infected cells [10]. Interestingly, EBV-associated cancers, such as gastric cancer and nasopharyngeal carcinomas, were characterised by the CpG methylator phenotype [12,13]. It was observed that global hypomethylation in the non-target tissue increased the risk of HNSCC [14]. Moreover, demethylation is a promising therapy for HPV-positive HNSCC [15,16]. Therefore, it appears that the analysis of methylation levels is necessary to improve the cancer therapies and prognoses.

We examined global DNA methylation levels in the tumour and the margin samples in OPSCC and OSCC in relation to the presence of HPV and EBV. Moreover, an analysis of global DNA methylation and the clinical–pathological and socio-demographic data was conducted.

## 2. Materials and Methods

### 2.1. Patients and Sample Collection

The study comprised 20 cases with OPSCC and 39 patients with OSCC. All the patients were recruited from the Department of Otorhinolaryngology and Oncological Laryngology in Zabrze, Medical University of Silesia in Katowice between the years of 2016 and 2022. The inclusion criteria were primary OPSCC or OSCC, age over 18 years, and written informed consent to participate in the study. The exclusion criteria were preoperative chemotherapy, radiotherapy, and confirmed systemic diseases. All the tumour and histologically normal margin samples were obtained during surgical resection. The tumour samples were histologically diagnosed as primary OPSCC or OSCC, while the margin samples were histologically confirmed to be free of cancer cells and dysplasia. The tumour stage was determined according to the TNM classification of the International Union Against Cancer (UICC) classification of head and neck tumours (7th Edition) [17]. The study group’s data, such as age, sex, and smoking and drinking habits, were collected using a questionnaire. The samples from all the groups were stored at −80 °C until further analysis. All molecular analyses were performed at the Department of Medical and Molecular Biology, Faculty of Medical Sciences in Zabrze, Medical University of Silesia. 

The study was approved by the Bioethics Committee of the Medical University of Silesia (No. KNW/0022/KB1/49/16 and No. KNW/0022/KB1/49/II/16/17). All the volunteers gave their written informed consent to participate in the study. 

### 2.2. DNA Extraction

The homogenisation of the tissue samples made use of Lysing Matrix A tubes (MP Biomedicals, Irvine, CA, USA) in a FastPrep^®^-24 instrument (MP Biomedicals, USA). DNA isolation from all the tumour and margin samples was conducted using a Gene Matrix Tissue DNA Purification Kit (EURx, Gdańsk, Poland) according to the manufacturers’ instructions. The concentration and purity of the isolated DNA were measured spectrophotometrically with a NanoPhotometer^®^ Pearl spectrophotometer (IMPLEN, München, Germany).

### 2.3. Global DNA Methylation Analysis

The global methylation levels were assessed by the enzyme-linked immunosorbent assay (ELISA) in the previously isolated DNA. We used the 5-mC DNA ELISA Kit (Zymo Research, Irvine, CA, USA) according to the manufacturers’ protocol. The commercial kit included a unique Anti-5-Methylcytosine monoclonal antibody, sensitive and specific for 5-mC. The percentage of 5-mC in the DNA samples could be accurately quantified from a standard curve. The standard curve was constructed with the negative and positive controls which were used to prepare seven standards. The final methylation concentrations were 0%, 5%, 10%, 25%, 50%, 75%, and 100%. One hundred nanograms (ng) of each DNA sample was used for the analysis, while all the samples were analysed in duplicate. An ELISA plate reader (BioTek Instruments, Inc., Winooski, VT, USA) was used to measure the absorbance at 405 nm. The percentage of 5-mC for DNA samples was calculated using the following logarithmic equation: % 5-mC = e{(Absorbance—y-intercept)Slope}.

### 2.4. Detection of HPV and EBV

The HPV and EBV infection status of the cohort was obtained from our previous studies [18,19]. HPV detection was conducted by hybridisation with the use of GenoFlow HPV Array Test Kits (DiagCor Bioscience Inc., Hong Kong, China) [18]. EBV detection was performed using real-time PCR with an EBV PCR Kit (EBV/ISEX/100, GeneProof, Brno, Czech Republic) [19].

### 2.5. Statistical Analysis

The statistical analysis used STATISTICA v. 13.36.0 software (StatSoft, Krakow, Poland) and R version 4.2.2 with the stats [20], survival [21,22], survminer [23], and dplyr [24] packages in RStudio version 2022.12.0 build 353 (PBC, Boston, MA, USA). Comparisons between global DNA methylation levels in the study groups and their detailed characteristics (age, sex, smoking, drinking, TNM, grade, and survival) were carried out using the Kruskal–Wallis test. The data were presented as mean values ± standard deviation (SD). The Fisher exact test was used to compare the groups based on sex, smoking, alcohol consumption, tumour location, HPV status, EBV status, and coinfection of HPV and EBV. The level of statistical significance was set at 0.05. The Spearman rank correlation coefficient (r_S_) was calculated to assess correlations between T, N, and the histological grade with global DNA methylation in the OPSCC and OSCC groups, and the correlation between age and global DNA methylation was evaluated in all groups. Three-year overall survival data were analysed using the Cox proportional hazards model and Kaplan–Meier curve.

## 3. Results

### 3.1. Study Groups

The OPSCC group included 20 patients (mean age 62.20 ± 8.15): 5 (25%) women and 15 (75%) men. The mean survival for OPSCC patients was 827.5 ± 465.05 days, and the overall 3-year survival rate was 70%. The OSCC group comprised 39 patients (mean age 58.41 ± 10.93) and included 14 (35.9%) women and 25 (64.1%) men. The survival analysis of the OSCC patients pointed to the overall 3-year survival rate of 45% and the mean survival time of 620.79 ± 460.63 days. The detailed characteristics of the study groups are presented in Table 1.

### 3.2. Global DNA Methylation Levels in OPSCC in Relation to the Socio-Demographic and Clinical–Pathological Parameters and the Presence of HPV and EBV

No significant difference in global DNA methylation was observed between the OPSCC tumours and the margin samples (*p* = 0.534). However, the OPSCC tumour samples obtained from women showed lower global DNA methylation levels than those from men (1.3% ± 0.99% vs. 3.5% ± 3.0%, *p* = 0.049). The OPSCC patients with HPV and EBV coinfection showed global DNA methylation that was lower in the margin samples than those free of coinfection (*p* = 0.042) (Table 2). The global DNA methylation levels in the tumour specimens correlated positively with the global DNA methylation levels in the margin samples (r_S_ = 0.59, *p* = 0.007). 

No association was detected between the global methylation, smoking, drinking, HPV and EBV infection, T classification, nodal status, histological grade, or 3-year survival. Moreover, we found no statistical differences in the socio-demographic or the clinical–pathological parameters or the presence of HPV and EBV in the tumour samples, as compared to the margin samples.

### 3.3. Global DNA Methylation Levels in OSCC in Relation to the Socio-Demographic and Clinical–Pathological Parameters and the Presence of HPV and EBV

No significant difference in global methylation levels was found between the OSCC tumours and the margin samples (*p* = 0.451). G3 tumours from OSCC patients had significantly lower levels of global DNA methylation than G2 tumours (0.98% ± 0.74% vs. 3.77% ± 4.97%, *p* = 0.010). Additionally, tumours from HPV-positive OSCC patients had significantly lower global DNA methylation levels than those from HPV-negative individuals (*p* = 0.013) (Table 3). 

A significant negative correlation between global DNA methylation and the N stage of OSCC patients (r_S_ = −0.33, *p* = 0.039) was observed in the margin samples. No significant correlation was observed between the tumour and the margin samples in the OSCC group (r_S_ = 0.25, *p* = 0.119).

No significant differences were detected in the global methylation levels, sex, smoking and drinking, HPV and EBV infection, T classification, nodal status, histological grade, and 3-year survival. Moreover, we found no statistical association between the 3-year survival, socio-demographic and clinical–pathological parameters, and the presence of HPV and EBV in the tumour samples, as compared to the margin samples.

### 3.4. Comparison of OPSCC and OSCC Samples

No significant difference was detected in the global methylation levels between the OPSCC and OSCC tumour samples (*p* = 0.313) or the margin samples (*p* = 0.366).

We found significantly higher levels of global DNA methylation in the tumour samples from HPV-positive OPSCC patients than in those from HPV-positive OSCC patients (3.2% ± 3.26% vs. 0.92% ± 0.83%; *p* = 0.015). Similarly, HPV-16-positive OPSCC patients had higher global DNA methylation levels in the tumour samples than HPV-16-positive OSCC cases (3.61% ± 3.4% vs. 1.07% ± 0.85%; *p* = 0.021). No other differences in global methylation levels were observed between OPSCC and OSCC individuals and between the margin samples in both groups.

## 4. Discussion

DNA methylation is one of the well-studied epigenetic modifications, often altered in cancer cells [25]. Changes in DNA methylation are analysed by different methods: global DNA methylation is used to assess the total genomic amount of 5-mC, while the genome-wide DNA methylation profile is used to analyse the DNA methylation of CpG located throughout the whole genome or only in selected loci or regions [26]. 

The present study analysed global DNA methylation levels in the tumour and margin samples collected from OPSCC and OSCC patients in relation to the socio-demographic and clinical–pathological parameters and the presence of HPV and EBV. No significant correlations were found between global methylation and the clinical–pathological parameters in the OPSCC samples. However, in the margin samples of OSCC patients, we found a significant negative correlation between global DNA methylation and the N stage. Furthermore, it was found that G3 tumours from OSCC patients showed significantly lower levels of global DNA methylation than G2 tumours from OSCC patients (*p* = 0.010). Some studies found no correlation between global DNA methylation and OSCC stage, location, or the histological grade [27,28], while Smith et al. [29] indicate that the degree of the hypomethylation of long interspersed nuclear element (LINE) sequences increased with the tumour stage, suggesting that methylation decreased as the tumour progressed in HNSCC patients [29].

We observed a connection between sex and the level of global DNA methylation in OPSCC patients. The methylation levels in women were significantly lower than in men (1.3% vs. 3.52%). Differences between methylation and sex were also observed in tongue cancer. Chen et al. [27] showed that hypomethylation in TSCC tissue was associated with the female sex. Also, Hsiung et al. [14] found reduced global DNA methylation in women. On the other hand, some studies found no significant differences in methylation profiles between sexes [28,29]. The differences between methylation profiles in women may be associated with folate and other nutrients involved in one-carbon metabolism, where folate and the methionine cycle generate a methyl donor (S-adenosylmethionine) involved in methylation reactions [30]. Moreover, folate is necessary for the maintenance of erythropoiesis [31]. Zhang et al. [32] observed reduced global methylation in cancer-free patients and suggested that menstruation could affect the smaller amount of folic acid necessary for one-carbon metabolism. Interestingly, perfluoroalkyl substances (water contaminants) may also influence decreased methylation in differentially methylated positions (DMPs) [33].

Our study found no significant DNA methylation differences between the tumour and margin samples in each of the study groups. In the present analysis, the margin samples were considered free of tumours and dysplasia by histopathologists. Of note, the term “carcinogenic field” indicates ongoing genetic and epigenetic molecular changes in the tissue surrounding the tumour, which is assessed microscopically as free from any pathological changes. In turn, the term “minimal residual cancer” refers to cancer cells remaining in the tissue after the surgical removal of the tumour. Such cells are not detected by routine diagnostic methods and may contribute to recurrence [34] and affect the methylation profile in the negative margin [35]. The carcinogenic field and the minimal residual cancer in the tissue may also influence the epigenetic changes in the margin, which is defined by histopathologists as a non-cancer sample.

Our study found that HPV-positive OPSCC patients had global DNA methylation higher than HPV-positive OSCC patients. This difference in the methylation level could be related to sample localisation due to the tissue-specific nature of the methylation profile or different pathomechanisms of carcinogenesis between OPSCC and OSCC. On the other hand, the comparison of HPV-positive and HPV-negative OPSCC patients revealed no significant differences in the global methylation levels. However, in the OPSCC patients with coinfections of HPV and EBV, lower global DNA methylation was observed, as compared to patients without HPV and EBV. Furthermore, we reported significantly lower global methylation levels in the OSCC tumours obtained from HPV-positive OSCC patients, as compared to HPV-negative OSCC individuals. Different methylation profiles were observed between HPV-positive and HPV-negative HNSCC patients [33,36,37,38,39]. Hinić et al. [40] observed that the overall median methylation levels between HPV-positive and HPV-negative HNSCC patients were comparable, which is in line with our findings in the OPSCC group. Also, Basu et al. [41] noted no significant differences in the methylation levels of DMPs between HPV-positive and HPV-negative OSCC patients. On the other hand, long interspersed nuclear elements 1 (LINE-1) had higher methylation levels in HPV-positive HNSCC patients than in HPV-negative HNSCC ones [42]. Additionally, LINE-1 showed higher methylation levels in HPV or HPV-16 positive OPSCC patients, compared to patients without HPV infection [43,44,45]. The hypermethylation of other transposable elements was more common in HPV-positive OPSCC patients [43]. Furthermore, Nakagawa et al. [46], who analysed the DNA methylome using microarrays, observed higher- and intermediate-methylation epigenotypes in HPV-associated OPSCC samples, while the low-methylation epigenotype was found mainly in HPV-negative OPSCC. Esposti et al. [37] carried out a detailed analysis of the methylation pattern to reveal both the hypo- and hyper-methylation of various genomic regions, corresponding to HPV infection, tumour location, and origin tissue as well as demographic characteristics. A more recent study of methylation sequencing by Rivera-Peña et al. [47] suggests that *PAX1* gene methylation differs between various HNSCC anatomic sites, and irregular DNA methylation patterns can be seen in the oral cavity, pharynx, and larynx subsites of HNSCC tumours. Moreover, as shown by Zygouras et al. [48], even the methylation of the specific regions of the *L1* HPV-16 gene has an impact on HNSCC tumour differentiation. Making use of the global DNA methylation, our study cannot be related to those results due to its limitations; however, it can provide an insight into the general methylome differences between OSCC and OPSCC tumours.

Some studies showed aberrant promoter methylation in cell lines and the samples of HNSCC patients, including nasopharyngeal carcinoma (NPC) in relation to EBV status [49,50]. The hypermethylation of the promoter regions occurs frequently in EBV-positive OSCC [51]. Moreover, differences in the methylation patterns were observed in NPC patients in relation to EBV status. Interestingly, EBV-negative NPC patients had methylation profiles similar to HNSCC patients [49]. Also, the comparison between EBV-negative, non-cancerous nasopharyngeal epithelial samples and EBV-positive NPC samples showed different methylation profiles [52]. The above studies presented different methylation profiles, which could be related to the choice of methods and the sequences. However, most of them showed the interdependence between viruses and the methylation profile. It is worth underlining that viruses like HPV and EBV may impact the methylation level. DNA tumour viruses increase host DNA methylation to repress host immune-related genes, as well as decrease DNA methylation level and regulate the expression of the host gene [53].

The main limitations of the present study are related to the relatively small number of cancer samples. Therefore, further studies on larger cohorts are warranted. We plan to conduct further analyses on larger cohorts using a more detailed qualitative method of DNA methylation sequencing. Future studies will analyse the impact of the DNA methylation patterns on 5-year survival and recurrence and the impact of coexisting systemic diseases.

## 5. Conclusions

The level of global DNA methylation differs depending on viral factors and varies strongly between OPSCC and OSCC patients. Unravelling the complexity of the methylation pattern changes and their relation to various tumour locations, tumour tissues of origin, HPV and EBV infection and coinfection, and the demographic and clinical characteristics of patients presents a significant challenge. Further analyses of larger cohorts and studies of the methylation patterns making use of methylation sequencing are warranted to confirm our findings.

## Figures and Tables

**Table 1:** Characteristics of the study groups.

Parameter		OPSCC	OSCC
		*n* (%)	
Age	Mean ± SD	62.20 ± 8.15	58.41 ± 10.93
Sex	Women	5 (25)	14 (35.9)
	Men	15 (75)	25 (64.1)
Smoking	Yes	9 (45)	31 (79.49)
	No	11 (55)	8 (20.51)
Drinking	Yes	11 (55)	24 (61.54)
	No	9 (45)	15 (38.46)
Smoking and drinking	Yes	6 (30)	20 (51.28)
	No	14 (70)	19 (48.72)
HPV infection	Yes	12 (60)	10 (25.64)
	No	7 (35)	26 (66.67)
	Unknown	1 (5)	3 (7.69)
HPV-16 infection	Yes	10 (50)	8 (20.51)
	No	8 (40)	28 (71.79)
	Unknown	2 (10)	3 (7.69)
EBV infection	Yes	4 (20)	19 (48.72)
	No	9 (45)	18 (46.15)
	Unknown	7 (35)	2 (5.13)
Coinfection of HPV and EBV	Yes	3 (15)	1 (2.56)
	No	10 (50)	34 (87.18)
	Unknown	7 (35)	4 (10.26)
T classification	T1	4 (20)	7 (17.95)
	T2	7 (35)	17 (43.59)
	T3	8 (40)	8 (20.51)
	T4	1 (5)	7 (17.95)
	T1 + T2	11 (55)	24 (61.54)
	T3 + T4	9 (45)	15 (38.46)
Nodal status	N0	9 (45)	19 (48.72)
	N1	2 (10)	6 (15.38)
	N2	8 (40)	12 (30.77)
	N3	1 (5)	2 (5.13)
	N0 + N1	11 (55)	25 (64.10)
	N2 + N3	9 (45)	14 (35.90)
Histological grading	G1	4 (20)	6 (15.38)
	G2	9 (45)	24 (61.54)
	G3	7 (35)	9 (23.08)
Overall 3-year survival rate	%	70	45

**Table 2:** Global DNA methylation levels in OPSCC patients according to particular characteristics.

Parameters		Global DNA Methylation Level [%]					
		OPSCC Tumour			OPSCC Margin		
		Mean ± SD	95% CI	*p*	Mean ± SD	95% CI	*p*
Group		2.96 ± 2.82	1.65–4.28	-	2.20 ± 1.57	1.47–2.93	-
Sex	Women	1.3 ± 0.99	0.07–2.53	0.049	1.24 ± 0.82	0.22–2.25	0.081
	Men	3.52 ± 3.03	1.84–5.2		2.52 ± 1.64	1.61–3.43	
Smoking	Yes	2.34 ± 2.14	0.7–3.99	0.447	2.5 ± 1.55	1.31–3.69	0.323
	No	3.47 ± 3.29	1.26–5.68		1.95 ± 1.61	0.87–3.03	
Drinking	Yes	3.32 ± 3.15	1.2–5.43	0.403	2.14 ± 1.78	0.95–3.34	0.790
	No	2.53 ± 2.46	0.64–4.43		2.27 ± 1.36	1.22–3.32	
Smoking and drinking	Yes	2.91 ± 2.4	0.39–5.43	0.741	2.41 ± 1.66	0.67–4.15	0.592
	No	2.99 ± 3.06	1.22–4.76		2.11 ± 1.58	1.2–3.02	
HPV infection	Yes	3.2 ± 3.26	1.13–5.27	0.767	2.11 ± 1.63	1.08–3.15	0.672
	No	2.58 ± 2.33	0.43–4.73		2.59 ± 1.5	1.21–3.98	
HPV-16 infection	Yes	3.61 ± 3.4	1.17–6.04	0.423	2.37 ± 1.62	1.21–3.53	0.859
	No	2.54 ± 2.16	0.74–4.34		2.48 ± 1.42	1.29–3.67	
EBV infection	Yes	1.26 ± 1.09	−0.47–3	0.279	1.01 ± 1.16	−0.85–2.86	0.076
	No	2.93 ± 3.17	0.49–5.36		2.7 ± 1.56	1.5–3.91	
Coinfection of HPV and EBV	Yes	1.34 ± 1.32	−1.95–4.62	0.498	0.63 ± 1.09	−2.08–3.35	0.042
	No	2.74 ± 3.05	0.55–4.92		2.65 ± 1.49	1.58–3.71	
Overall 3-year survival	Yes	2.5 ± 2.0	1.3–3.7	0.71	2.0 ± 1.3	1.2–2.8	0.62
	No	4.0 ± 4.2	−0.4–8.4		2.7 ± 2.1	0.5–4.9	

SD—standard deviation.

**Table 3:** Global DNA methylation levels in OSCC patients according to particular characteristics.

Parameters		Global DNA Methylation Level [%]					
		OSCC Tumour			OSCC Margin		
		Mean ± SD	95% CI	*p*	Mean ± SD	95% CI	*p*
Group		3.27 ± 4.51	1.81–4.74	-	2.45 ± 3.54	1.30–3.59	-
Sex	Women	3.74 ± 4.91	0.91–6.58	0.736	3.26 ± 5.15	0.28–6.23	0.661
	Men	3.01 ± 4.35	1.22–4.81		1.99 ± 2.2	1.08–2.9	
Smoking	Yes	3.29 ± 4.81	1.53–5.06	0.531	2.33 ± 3.79	0.94–3.73	0.099
	No	3.19 ± 3.31	0.42–5.96		2.88 ± 2.43	0.84–4.91	
Drinking	Yes	3.71 ± 5.26	1.49–5.93	0.718	2.22 ± 2.61	1.12–3.32	0.644
	No	2.57 ± 2.98	0.93–4.22		2.81 ± 4.74	0.18–5.43	
Smoking and drinking	Yes	3.51 ± 5.51	0.93–6.09	0.736	2.05 ± 2.45	0.91–3.2	0.491
	No	3.02 ± 3.27	1.45–4.6		2.86 ± 4.44	0.72–5	
HPV infection	Yes	0.92 ± 0.83	0.32–1.51	0.013	1.91 ± 1.77	0.65–3.18	0.778
	No	3.55 ± 3.99	1.94–5.16		2.51 ± 3.91	0.93–4.09	
HPV-16 infection	Yes	1.07 ± 0.85	0.36–1.79	0.098	1.75 ± 1.4	0.58–2.92	0.849
	No	3.32 ± 3.93	1.79–4.84		2.51 ± 3.83	1.03–4	
EBV infection	Yes	4.02 ± 4.99	1.61–6.43	0.145	3.2 ± 4.82	0.88–5.52	0.693
	No	2.79 ± 4.16	0.72–4.86		1.59 ± 1.16	1.02–2.17	
Coinfection of HPV and EBV	Yes	0.04	-	0.137	0.32	-	0.198
	No	2.97 ± 3.65	1.69–4.24		2.32 ± 3.49	1.1–3.54	
Overall 3-year survival	Yes	4.0 ± 5.9	1.0–7.1	0.941	2.7 ± 2.8	1.3–4.2	0.207
	No	2.8 ± 3.1	1.4–4.2		2.3 ± 4.2	0.4–4.2	

SD—standard deviation.

## Data Availability

The data used to support the findings of this study are available from the corresponding author upon request.

## References

[1] Moore L., Le T., Fan G. (2013). DNA Methylation and Its Basic Function. Neuropsychopharmacology.

[2] Weisenberger D.J. (2014). Characterizing DNA methylation alterations from The Cancer Genome Atlas. J. Clin. Investig..

[3] Mikeska T., Craig J.M. (2014). DNA methylation biomarkers: Cancer and beyond. Genes.

[4] Johnson D.E., Burtness B., Leemans C.R., Lui V.W.Y., Bauman J.E., Grandis J.R. (2020). Head and neck squamous cell carcinoma. Nat. Rev. Dis. Primers.

[5] Gormley M., Creaney G., Schache A., Ingarfield K., Conway D.I. (2022). Reviewing the epidemiology of head and neck cancer: Definitions, trends and risk factors. Br. Dent. J..

[6] Warnakulasuriya S. (2009). Global epidemiology of oral and oropharyngeal cancer. Oral. Oncol..

[7] Carlander A.F., Jakobsen K.K., Bendtsen S.K., Garset-Zamani M., Lynggaard C.D., Jensen J.S., Grønhøj C., Buchwald C.V. (2021). A Contemporary Systematic Review on Repartition of HPV-Positivity in Oropharyngeal Cancer Worldwide. Viruses.

[8] Lechner M., Liu J., Masterson L., Fenton T.R. (2022). HPV-associated oropharyngeal cancer: Epidemiology, molecular biology and clinical management. Nat. Rev. Clin. Oncol..

[9] Nakagawa T., Kurokawa T., Mima M., Imamoto S., Mizokami H., Kondo S., Okamoto Y., Misawa K., Hanazawa T., Kaneda A. (2021). DNA Methylation and HPV-Associated Head and Neck Cancer. Microorganisms.

[10] Pei Y., Wong J.H., Robertson E.S. (2020). Herpesvirus Epigenetic Reprogramming and Oncogenesis. Annu. Rev. Virol..

[11] She Y., Nong X., Zhang M., Wang M. (2017). Epstein-Barr virus infection and oral squamous cell carcinoma risk: A meta-analysis. PLoS ONE.

[12] Stanland L.J., Luftig M.A. (2020). The Role of EBV-Induced Hypermethylation in Gastric Cancer Tumorigenesis. Viruses.

[13] Chow L.K.-Y., Chung D.L.-S., Tao L., Chan K.F., Tung S.Y., Ngan R.K.C., Ng W.T., Lee A.W.-M., Yau C.C., Kwong D.L.-W. (2022). Epigenomic landscape study reveals molecular subtypes and EBV-associated regulatory epigenome reprogramming in na-sopharyngeal carcinoma. EBioMedicine.

[14] Hsiung D.T., Marsit C.J., Houseman E.A., Eddy K., Furniss C.S., McClean M.D., Kelsey K.T. (2007). Global DNA methylation level in whole blood as a biomarker in head and neck squamous cell carcinoma. Cancer Epidemiol. Biomark. Prev..

[15] Biktasova A., Hajek M., Sewell A., Gary C., Bellinger G., Deshpande H.A., Bhatia A., Burtness B., Judson B., Mehra S. (2017). Demethylation Therapy as a Targeted Treatment for Human Papillomavirus-Associated Head and Neck Cancer. Clin. Cancer Res..

[16] Hajek M., Biktasova A., Sewell A., Gary C., Cantalupo P., Anderson K.S., Yarbrough W.G., Issaeva N. (2020). Global Genome Demethylation Causes Transcription-Associated DNA Double Strand Breaks in HPV-Associated Head and Neck Cancer Cells. Cancers.

[17] Sobin L.H., Gospodarowicz M.K., Wittekind C. (2010). TNM Classification of Malignant Tumours.

[18] Strzelczyk J.K., Biernacki K., Gaździcka J., Chełmecka E., Miśkiewicz-Orczyk K., Zięba N., Strzelczyk J., Misiołek M. (2021). The Prevalence of High- and Low-Risk Types of HPV in Patients with Squamous Cell Carcinoma of the Head and Neck, Patients with Chronic Tonsillitis, and Healthy Individuals Living in Poland. Diagnostics.

[19] Strzelczyk J.K., Świętek A., Biernacki K., Gołąbek K., Gaździcka J., Miśkiewicz-Orczyk K., Ścierski W., Strzelczyk J., Fiolka R., Misiołek M. (2022). PCR Detection of Epstein-Barr Virus (EBV) DNA in Patients with Head and Neck Squamous Cell Carcinoma, in Patients with Chronic Tonsillitis, and in Healthy Individuals. Biomed. Res. Int..

[20] R Core Team R: A Language and Environment for Statistical Computing. https://www.r-project.org/.

[21] Therneau T. A Package for Survival Analysis in R [R Package Version 3.5-5]. https://cran.r-project.org/package=survival.

[22] Therneau T.M., Grambsch P.M. (2000). Modeling Survival Data: Extending the Cox Model.

[23] Kassambara A., Kosinski M., Biecek P. Survminer: Drawing Survival Curves Using g‘gplot2’ [R Package Version 0.4.9]. https://CRAN.R-project.org/package=survminer.

[24] Wickham H., François R., Henry L., Müller K., Vaughan D. dplyr: A Grammar of Data Manipulation [R Package Version 1.1.4]. https://CRAN.R-project.org/package=dplyr.

[25] Skvortsova K., Stirzaker C., Taberlay P. (2019). The DNA methylation landscape in cancer. Essays Biochem..

[26] Martisova A., Holcakova J., Izadi N., Sebuyoya R., Hrstka R., Bartosik M. (2021). DNA Methylation in Solid Tumors: Functions and Methods of Detection. Int. J. Mol. Sci..

[27] Chen H.C., Yang C.M., Cheng J.T., Tsai K.W., Fu T.Y., Liou H.H., Tseng H.H., Lee J.H., Li G.C., Wang J.S. (2016). Global DNA hypomethylation is associated with the development and poor prognosis of tongue squamous cell carcinoma. J. Oral. Pathol. Med..

[28] Subbalekha K., Pimkhaokham A., Pavasant P., Chindavijak S., Phokaew C., Shuangshoti S., Matangkasombut O., Mutirangura A. (2009). Detection of LINE-1s hypomethylation in oral rinses of oral squamous cell carcinoma patients. Oral. Oncol..

[29] Smith I.M., Mydlarz W.K., Mithani S.K., Califano J.A. (2007). DNA global hypomethylation in squamous cell head and neck cancer associated with smoking, alcohol consumption and stage. Int. J. Cancer.

[30] Amenyah S.D., Hughes C.F., Ward M., Rosborough S., Deane J., Thursby S.J., Walsh C.P., Kok D.E., Strain J.J., McNulty H. (2020). Influence of nutrients involved in one-carbon metabolism on DNA methylation in adults-a systematic review and meta-analysis. Nutr. Rev..

[31] Khan K.M., Jialal I. Folic Acid Deficiency—StatPearls—NCBI [Updated 2022 June 27]. https://www.ncbi.nlm.nih.gov/books/NBK535377/.

[32] Zhang F.F., Cardarelli R., Carroll J., Fulda K.G., Kaur M., Gonzalez K., Vishwanatha J.K., Santella R.M., Morabia A. (2011). Significant differences in global genomic DNA methylation by gender and race/ethnicity in peripheral blood. Epigenetics.

[33] Xu Y., Jurkovic-Mlakar S., Lindh C.H., Scott K., Fletcher T., Jakobsson K., Engström K. (2020). Associations between serum concentrations of perfluoroalkyl substances and DNA methylation in women exposed through drinking water: A pilot study in Ronneby, Sweden. Environ. Int..

[34] Pierik A.S., Leemans C.R., Brakenhoff R.H. (2021). Resection Margins in Head and Neck Cancer Surgery: An Update of Residual Disease and Field Cancerization. Cancers.

[35] Sorroche B.P., Talukdar F.R., Lima S.C.S., Melendez M.E., de Carvalho A.C., de Almeida G.C., De Marchi P., Lopes M., Ribeiro Pinto L.F., Carvalho A.L. (2021). DNA Methylation Markers from Negative Surgical Margins Can Predict Recurrence of Oral Squamous Cell Carcinoma. Cancers.

[36] Cancer Genome Atlas Network (2015). Comprehensive genomic characterization of head and neck squamous cell carcinomas. Nature.

[37] Degli Esposti D., Sklias A., Lima S.C., Beghelli-de la Forest Divonne S., Cahais V., Fernandez-Jimenez N., Cros M.P., Ecsedi S., Cuenin C., Bouaoun L. (2017). Unique DNA methylation signature in HPV-positive head and neck squamous cell car-cinomas. Genome Med..

[38] Berglund A., Muenyi C., Siegel E.M., Ajidahun A., Eschrich S.A., Wong D., Hendrick L.E., Putney R.M., Kim S., Hayes D.N. (2022). Characterization of Epigenomic Alterations in HPV16+ Head and Neck Squamous Cell Carcinomas. Cancer Epidemiol. Biomarks Prev..

[39] Worsham M.J., Chen K.M., Ghanem T., Stephen J.K., Divine G. (2013). Epigenetic modulation of signal transduction pathways in HPV-associated HNSCC. Otolaryngol. Head Neck Surg..

[40] Hinić S., Rich A., Anayannis N.V., Cabarcas-Petroski S., Schramm L., Meneses P.I. (2022). Gene Expression and DNA Methylation in Human Papillomavirus Positive and Negative Head and Neck Squamous Cell Carcinomas. Int. J. Mol. Sci..

[41] Basu B., Chakraborty J., Chandra A., Katarkar A., Baldevbhai J.R.K., Dhar Chowdhury D., Ray J.G., Chaudhuri K., Chatterjee R. (2017). Genome-wide DNA methylation profile identified a unique set of differentially methylated immune genes in oral squamous cell carcinoma patients in India. Clin. Epigenetics.

[42] Richards K.L., Zhang B., Baggerly K.A., Colella S., Lang J.C., Schuller D.E., Krahe R. (2009). Genome-wide hypomethylation in head and neck cancer is more pronounced in HPV-negative tumors and is associated with genomic instability. PLoS ONE.

[43] Camuzi D., Buexm L.A., Lourenço S.Q.C., Esposti D.D., Cuenin C., Lopes M.S.A., Manara F., Talukdar F.R., Herceg Z., Ribeiro Pinto L.F. (2021). HPV Infection Leaves a DNA Methylation Signature in Oropharyngeal Cancer Affecting Both Coding Genes and Transposable Elements. Cancers.

[44] Furlan C., Polesel J., Barzan L., Franchin G., Sulfaro S., Romeo S., Colizzi F., Rizzo A., Baggio V., Giacomarra V. (2017). Prognostic significance of LINE-1 hypomethylation in oropharyngeal squamous cell carcinoma. Clin. Epigenetics.

[45] Casarotto M., Lupato V., Giurato G., Guerrieri R., Sulfaro S., Salvati A., D’Angelo E., Furlan C., Menegaldo A., Baboci L. (2022). LINE-1 hypomethylation is associated with poor outcomes in locoregionally advanced oropharyngeal cancer. Clin. Epigenetics.

[46] Nakagawa T., Matsusaka K., Misawa K., Ota S., Fukuyo M., Rahmutulla B., Kunii N., Sakurai D., Hanazawa T., Matsubara H. (2020). Stratification of HPV-associated and HPV-negative oropharyngeal squamous cell carcinomas based on DNA methylation epigenotypes. Int. J. Cancer.

[47] Rivera-Peña B., Folawiyo O., Turaga N., Rodríguez-Benítez R.J., Felici M.E., Aponte-Ortiz J.A., Pirini F., Rodríguez-Torres S., Vázquez R., López R. (2024). Promoter DNA methylation patterns in oral, laryngeal and oropharyngeal anatomical regions are associated with tumor differentiation, nodal involvement and survival. Oncol. Lett..

[48] Zygouras I., Leventakou D., Pouliakis A., Panagiotou S., Tsakogiannis D., Konstantopoulos G., Logotheti E., Samaras M., Kyriakopoulou Z., Beloukas A. (2023). Human Papillomavirus 16 DNA Methylation Patterns and Investigation of Integration Status in Head and Neck Cancer Cases. Int. J. Mol. Sci..

[49] Ooft M.L., van Ipenburg J., van Loo R., de Jong R., Moelans C., Braunius W., de Bree R., van Diest P., Koljenović S., Baatenburg de Jong R. (2018). Molecular profile of nasopharyngeal carcinoma: Analysing tumour sup-pressor gene promoter hypermethylation by multiplex ligation-dependent probe amplification. J. Clin. Pathol..

[50] Shi F., Zhou M., Shang L., Du Q., Li Y., Xie L., Liu X., Tang M., Luo X., Fan J. (2019). EBV(LMP1)-induced metabolic reprogramming inhibits necroptosis through the hypermethylation of the *RIP3* promoter. Theranostics.

[51] Burassakarn A., Pientong C., Sunthamala N., Chuerduangphui J., Vatanasapt P., Patarapadungkit N., Kongyingyoes B., Ekalaksananan T. (2017). Aberrant gene promoter methylation of E-cadherin, *p16 ^INK4a^*, *p14 ^ARF^*, and *MGMT* in Epstein-Barr virus-associated oral squamous cell carcinomas. Med. Oncol..

[52] Zhao W., Mo Y., Wang S., Midorikawa K., Ma N., Hiraku Y., Oikawa S., Huang G., Zhang Z., Murata M. (2017). Quantitation of DNA methylation in Epstein-Barr virus-associated nasopharyngeal carcinoma by bisulfite amplicon sequencing. BMC Cancer.

[53] Kuss-Duerkop S.K., Westrich J.A., Pyeon D. (2018). DNA Tumor Virus Regulation of Host DNA Methylation and Its Implications for Immune Evasion and Oncogenesis. Viruses.

